# Postoperative pain assessment of robotic nipple-sparing mastectomy with immediate prepectoral prosthesis breast reconstruction: a comparison with conventional nipple-sparing mastectomy

**DOI:** 10.7150/ijms.56997

**Published:** 2021-04-17

**Authors:** Jiae Moon, Jeea Lee, Dong Won Lee, Hye Sun Lee, Da Jung Nam, Min Jung Kim, Na Young Kim, Hyung Seok Park

**Affiliations:** 1 Department of Anesthesiology and Pain Medicine, Anesthesia and Pain Research Institute, Yonsei University College of Medicine, Seoul, Republic of Korea.; 2 Department of Surgery, Yonsei University College of Medicine, Seoul, Republic of Korea.; 3 Department of Plastic and Reconstructive Surgery, Yonsei University College of Medicine, Seoul, Republic of Korea.; 4 Department of Research Affairs, Biostatistics Collaboration Unit, Yonsei University College of Medicine, Seoul, Republic of Korea.; 5 Department of Anesthesiology and Pain Medicine, Anesthesia and Pain Research Institute, National Health Insurance Service Ilsan Hospital, 100 Ilsan-ro, Ilsandong-gu, Goyang, 10444, Republic of Korea.

**Keywords:** breast cancer, robotic, conventional, nipple-sparing mastectomy, postoperative pain

## Abstract

**Aims:** Nipple-sparing mastectomy (NSM) is a surgical procedure for patients with breast cancer without nipple-areolar complex (NAC) involvement. Robotic NSM (RNSM) with immediate breast reconstruction has been recently introduced; however, reports regarding RNSM are still lacking. Therefore, this study aimed to evaluate the postoperative assessment with a focus on postoperative pain of RNSM with prepectoral immediate prosthesis breast reconstruction (IPBR) compared with conventional NSM (CNSM) in patients with breast cancer without NAC invasion.

**Methods:** This retrospective study included 81 patients who underwent RNSM (n = 40) or CNSM (n = 41) with prepectoral IPBR using direct-to-implant or tissue expander between January 2018 and June 2020. The primary endpoint was to compare postoperative pain intensity based on a numerical rating scale (NRS). The secondary endpoint was to evaluate the postoperative recovery profile, including postoperative nausea/vomiting (PONV) and complications.

**Results:** A statistical difference was observed in the resting NRS scores at 0-6 postoperative hours between the RNSM and CNSM groups (3.2 ± 1.5 versus 4.2 ± 1.6, respectively; Bonferroni corrected *P* = 0.005), however, no difference was shown at other time periods. Also, no between-group difference was found in the NRS scores for acting pain within 48 postoperative hours and the number of patients requiring additional analgesics.

**Conclusions:** Despite a statistical difference in the resting NRS scores during the early postoperative phase, the absence of any significant difference in the requirement of additional analgesics between the groups suggested that RNSM does not significantly attenuate postoperative pain intensity.

## Introduction

Nipple-sparing mastectomy (NSM) is a surgical procedure performed in patients with breast cancer without the involvement of the nipple-areolar complex (NAC), to improve the cosmetic outcome and quality of life in women with early breast cancer or germline *BRCA1/2* mutation. In NSM, the skin envelope and NAC are preserved, and the glandular breast tissue is completely removed. NSM allows immediate breast reconstruction (IBR) using autologous tissue and/or implants 1. Preservation of the NAC enhances the esthetic outcome and patient satisfaction without compromising oncologic safety 2. However, dissecting and removing breast tissue through limited incisions is technically challenging. Some NSM incisions leave visible scars on the breast dome and cause distortion or malposition of the NAC. NAC-related necrosis also remains a major NSM complication 3,4.

Robotic NSM (RNSM) with IBR was first described by Toesca *et al*. 5 in October 2015 and has substantially contributed to overcoming the challenges of conventional NSM (CNSM) 6. A high-resolution ten-fold magnifying three-dimensional camera allows accurate visualization and better access to the surgical planes, which enables RNSM with IBR through a small axillary incision. The flexibility and sophisticated motion of robotic surgical systems increase the surgical accuracy in a limited space 3,7. RNSM with a 2.5-6-cm axillary incision has excellent patient satisfaction and cosmetic results, considering the absence of scarring in the anterior breast. RNSM has low ischemic NAC complication rates, which may be attributed to the remote incision from the NAC and the vascular advantage allowed by sparing small vessels responsible for vascular supply to the nipple 7,8.

Despite several limitations, including a longer duration of operation, higher cost, and lack of research on the long-term oncological outcomes, RNSM with IBR has been increasingly used worldwide given its definite advantage with respect to esthetics 6,8-11. However, there are few studies on the postoperative outcomes of RNSM with IBR 8.

Thus, this study aimed to evaluate the postoperative parameters, particularly postoperative pain, of RNSM with prepectoral immediate prosthesis breast reconstruction (IPBR) using direct-to-implant (DTI) or tissue expander, compared with CNSM in patients with breast cancer without NAC invasion.

## Materials and methods

### Patient population

**Figure 1:**
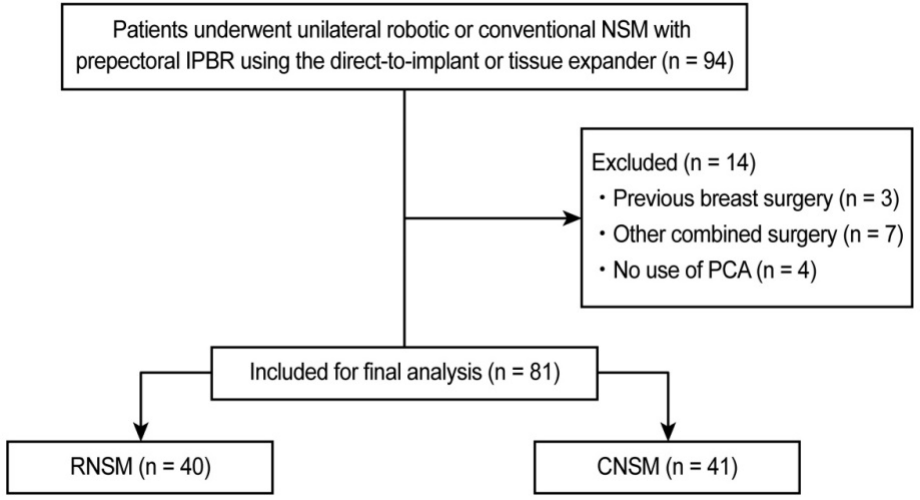
Consort flow diagram. NSM, nipple-sparing mastectomy; IPBR, immediate prosthesis breast reconstruction; PCA, patient-controlled analgesia; RNSM, robotic nipple-sparing mastectomy; CNSM, conventional nipple-sparing mastectomy.

We identified the records of 94 consecutive patients with breast cancer who underwent unilateral RNSM or CNSM with prepectoral IPBR using DTI or tissue expander between January 2018 and June 2020 at Severance Hospital, Seoul, Korea. Three patients who had previously undergone breast surgery, seven patients who underwent other simultaneous surgeries, and four patients who were not administered intravenous patient-controlled analgesia (IV-PCA) for postoperative pain control were excluded. Consequently, 81 patients who underwent RNSM (n = 40) and CNSM (n = 41) were analyzed (Figure 1).

### Surgical procedures

#### RNSM

Detailed procedures for RNSM have been previously described 3,9-11. Briefly, a 2.5-6-cm linear mid-axillary incision was made below the axillary fossa. The ipsilateral arm was straightened with internal rotation and abduction and fixed above the head. A sentinel lymph node biopsy was performed through the incision; moreover, the working space beneath the skin flap or retromammary space was manually developed. Subsequently, a single-port device was inserted through the same incision for the gas-inflated technique and the robotic surgical system was docked. After carbon dioxide gas insufflation with 8-10 mmHg for surgical field expander, the skin flap and/or retromammary space were dissected using the robotic surgical system. During the mastectomy, the sub-NAC tissue was resected to evaluate tumor cell involvement in a frozen section. Finally, the entire breast parenchyma was retrieved through the same incision, and prepectoral IPBR using DTI or tissue expander was performed by the plastic surgeons 3,10.

#### CNSM

Various skin incisions such as inframammary fold incision, radial incision, and/or periareolar/circumareolar incision were made. The skin flaps were developed along the superficial fascia superior to the lower clavicular border, medial to the ipsilateral sternal border, inferior to the rectus sheath, and posterior to the anterior latissimus dorsi muscle border. The entire breast gland was dissected from the pectoralis major muscle, including the pectoralis fascia. A frozen section for the sub-NAC tissue was intraoperatively obtained. Through the same incision, sentinel lymph node biopsy using navigator and/or axillary lymph node dissection was performed. After the mastectomy, prepectoral IPBR using tissue expander or implant was performed by the plastic surgeons 12.

#### IPBR

IPBR after mastectomy was performed using DTI or tissue expander in the prepectoral space. The implant or tissue expander was completely wrapped with acellular dermal matrix (ADM) before insertion. Patients undergoing autologous reconstruction or subpectoral prosthesis were excluded from the current study.

#### Anesthetic procedures

General anesthesia was administered following our institution's conventional protocol. After the patient arrived in the operating room, all devices monitoring oxygen saturation, electrocardiography, noninvasive arterial blood pressure, and bispectral index (BIS), were utilized to evaluate the patient. Intravenous (IV) injection of 0.1 mg glycopyrrolate was administered as premedication. Subsequently, anesthesia was induced using propofol (1-1.5 mg/kg), rocuronium (0.6 mg/kg), and remifentanil (0.05-0.1 μg/kg). Mechanical ventilation was initiated with an inspiration to expiration (I:E) ratio of 1:2, positive end-expiratory pressure of 5 cmH_2_O, tidal volume of 6-8 ml/kg, and respiratory rate of 8-14 frequency/min to maintain an end-tidal carbon dioxide of 35-42 mmHg. Anesthesia was maintained using sevoflurane or desflurane (0.8-1 age-adjusted minimum alveolar concentration) and remifentanil at 0.03-0.1 µg/kg/min. To maintain a constant anesthetic depth, the BIS was continuously monitored, with a target range of 40-60.

### Postoperative pain management

Prior to the end of the surgery, 1 μg/kg of fentanyl (Hana Pharm, Seoul, Korea) and 0.3 mg of ramosetron (Nasea®, Astellas Pharma Korea, Seoul, Korea) were concurrently administered to relieve postoperative pain and nausea/vomiting, respectively. All patients received an IV-PCA device (Anapa plus; E-HWA Biomedics, Seoul, Korea), programmed to 2 mL/h for background infusion, a demand volume of 0.5 mL, lock-out interval of 15 minutes, with a total volume of 100 mL. The PCA regimen comprised 15 ± 2 µg/kg of fentanyl and 0.3 mg of ramosetron, which were mixed with normal saline to achieve a total volume of 100 mL. Additionally, patients in both groups received 1 g IV paracetamol (profa^®^, Dai han Pharm, Seoul, Korea) at 8-h intervals for 5 days, and one Mypol^®^ tablet (codeine phosphate 10 mg plus ibuprofen 200 mg, Sung-won Adcock Pharm, Seoul, Korea) at 8-h intervals until discharge.

### Postoperative pain assessment

Data regarding postoperative pain was obtained from an electronic medical database, which was recorded by a PCA management team comprising two qualified nurses. Resting pain was defined as pain while at rest or staying still; acting pain was defined as pain during movement, posture change, or coughing. All eligible patients were informed on how to rate their pain intensity using the numerical rating scale (NRS; 0, no pain; 10, worst pain possible) in the pre-anesthetic room 13. After the patients were moved to the post-anesthetic care unit (PACU) and had emerged from anesthesia, the recovery nurses assessed their NRS scores. The patients were instructed about the use of the PCA device, and were encouraged to push the button whenever they experienced pain. In patients who experienced sustained pain with a resting NRS score ≥4, 50 µg IV fentanyl was administered as an additional rescue analgesic. After the patients were transferred to the admission room, postoperative resting and acting NRS score assessments were performed at 0-6, 6-24, and 24-48 postoperative hours. In patients who suffered from the prolonged pain with a resting NRS score of ≥4 in the admission room, 50 mg tridol (Tramadol HCL®, Yuhan. Co., Seoul, Korea) was administered as a rescue analgesic.

### Postoperative nausea and vomiting (PONV) and complications

Postoperative management during hospitalization was left at the discretion of the Yonsei Breast Cancer Center team. PONV was assessed on a 4-point NRS (0-3; 0 = none, 1 = mild, 2 = moderate, 3 = severe). All patients received 0.3 mg IV ramostron at 24-h intervals for 3 days. Rescue antiemetics were administered when severe nausea or vomiting developed, or upon request from the patients. Metoclopramide (Macperan®, Dong Wha Pharm. Co., Ltd., Seoul, Korea) 10 mg was administered as a first-line rescue antiemetic. Patients with persistent and refractory PONV received 0.3 mg IV ramosetron. The incidence of postoperative complications was assessed for up to 90 postoperative days. Major complications included implant loss, nipple necrosis, and/or mastectomy skin flap necrosis requiring surgical treatment. Mild complications included seroma, hematoma, minor infection, wound dehiscence, and nipple or skin flap necrosis with conservative management.

### Data collection

Demographic and preoperative characteristics, including age, body mass index (BMI), American Society of Anesthesiologists physical status, smoking history, menopause status, family history of breast cancer, and neoadjuvant chemotherapy were assessed. Regarding perioperative and surgical characteristics, duration of anesthesia and operation, blood loss, dose of remifentanil administered, dose of fentanyl combined with PCA, surgical incision length, mastectomy specimen weight, location, reconstruction type, reconstruction implant volume, and adjuvant treatment were assessed. Further, pathologic variables were collected. Additionally, postoperative profiles, including postoperative hospital stay, PONV, and complications were evaluated. Postoperative NRS scores to determine the maximum resting and acting pain were assessed in the PACU at 0-6, 6-24, and 24-48 postoperative hours. Furthermore, the number of patients requiring additional analgesics for up to postoperative 48 hours were assessed.

### Statistical analysis

Continuous variables are presented as mean ± standard deviation (SD) while categorical variables are presented as the number of patients (percentage). Between-group differences in the continuous and categorical variables were analyzed using the Student's t-test and the Chi-square test/Fisher's exact test, respectively. To determine the group and time effects for repeated-measure continuous and categorical variables, linear mixed model analysis and generalized estimating equations were performed, respectively. Bonferroni correction was applied to adjust for multiple comparisons in post-hoc analyses. P < 0.05 was considered statistically significant. All analyses were conducted using SAS version 9.4 (Cary, NC).

## Results

Table 1 demonstrates the clinicopathologic factors of the patients selected for this retrospective analysis. The average BMI in the RNSM group was lower than that in the CNSM group by 1.7 kg/m^2^ (22.2 ± 3.5 vs. 23.9 ± 3.6 kg/m^2^, *P* = 0.030), and no between-group differences were observed regarding other variables. Table 2 summarizes the intraoperative and surgical variables. Duration of anesthesia and operation were significantly longer in the RNSM group than in the CNSM group (both *P* < 0.001). There was no between-group difference in the dose of fentanyl used for PCA (15.0 ± 1.8 µg/kg, RNSM group; 14.6 ± 1.6 µg/kg, CNSM group). The surgical incision length in the RNSM group (4.4 ± 0.7 cm) was significantly shorter than that in the CNSM group (8.8 ± 2.2 cm; *P* <0.001).

**Figure 2:**
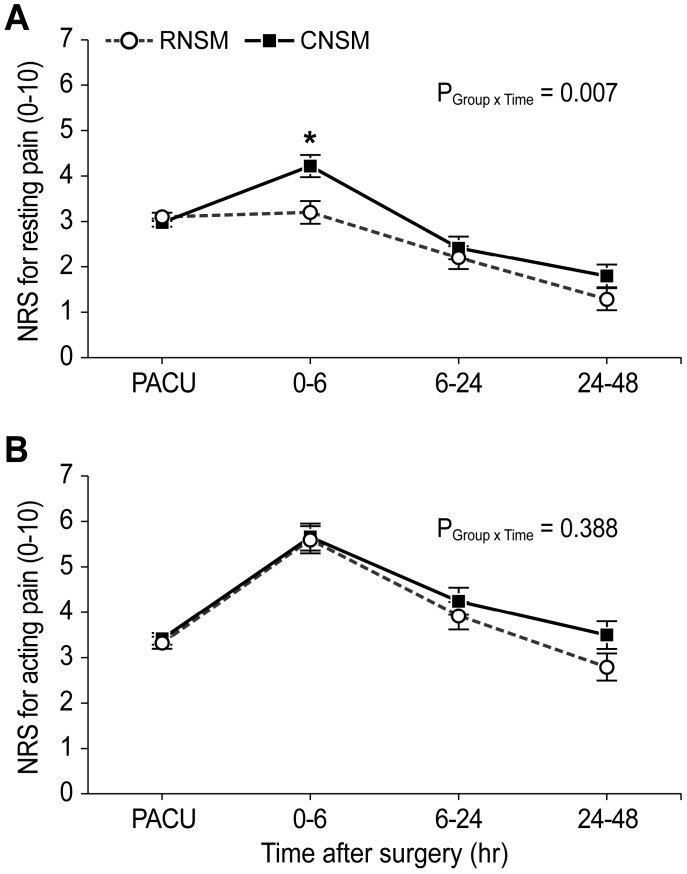
Resting (A) and acting (B) pain intensity in the RNSM and CNSM groups during 48 postoperative hours. NRS, numerical rating scale; RNSM, robotic nipple-sparing mastectomy; CNSM, conventional nipple-sparing mastectomy; PACU, post-anesthesia care unit. *Bonferroni corrected *P* = 0.005 compared with CNSM.

**Figure 3:**
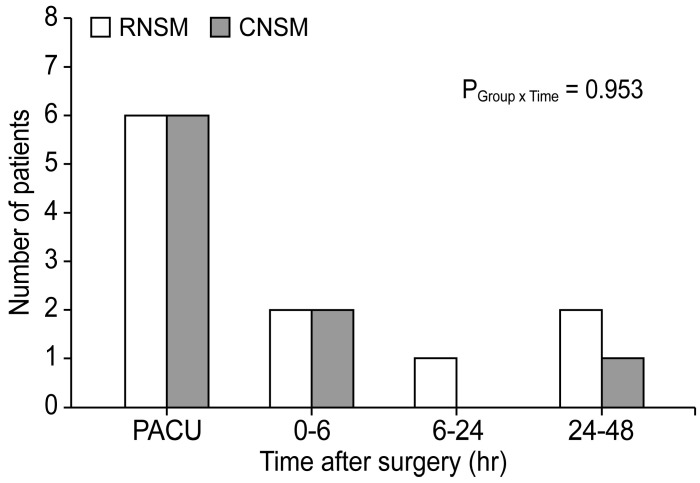
Number of patients who required additional analgesics during the first 48 postoperative hours. RNSM, robotic nipple sparing mastectomy; CNSM, conventional nipple sparing mastectomy; PACU, post-anesthesia care unit.

Figure 2 presents the resting and acting pain intensity. The linear mixed model analysis revealed significant between-group differences in the resting NRS score (P _Group x Time_ = 0.007) (Figure 2A). After the post-hoc analysis, a statistical difference was observed in the resting NRS scores at 0-6 postoperative hours between the RNSM and CNSM groups (3.2 ± 1.5 versus 4.2 ± 1.6, respectively; Bonferroni corrected *P* = 0.005); however, no difference was shown at other time periods (PACU, 6-24, and 24-48 postoperative hours). Additionally, there was no between-group difference in the NRS scores for acting pain up to 48 postoperative hours (Figure 2B), and in the number of patients requiring additional analgesics (Figure 3).

Table 3 presents the postoperative recovery profiles, including PONV and postoperative complications. In the analysis of the last 20 cases in each group, no difference was observed between the two groups in the average length of postoperative hospital stay (*P* = 0.252). Moreover, there was no between-group difference in the number of patients who had PONV and complications up to 90 postoperative days. No patient had unrecoverable complications.

## Discussion

This study analyzed postoperative pain in RNSM with prepectoral IPBR compared with CNSM, which showed a statistical difference in resting NRS scores between the two groups; however, the difference was transient and observed only during 0-6 postoperative hours. Further, no between-group difference was observed in the number of patients requiring additional analgesics, the incidence of PONV, and postoperative complications.

Approximately 25%-60% of patients present with persistent pain after breast cancer surgery, which is a major clinical issue 14,15. It leads to chronic pain in a substantial number of patients, which decreases the quality of life and deteriorates their physical functions 16,17. There may be multifactorial risk factors for this pain, including patient-related and treatment-related risk factors, such as the type of procedure performed 15. Therefore, there is a need for surgical approaches that alleviate postoperative pain to improve the postoperative outcome in patients. Minimally invasive robotic approaches have been demonstrated to reduce postoperative pain for various surgeries 18,19. Given the rapid expander of robotic approaches for breast procedures, there have been several reports regarding the feasibility and advantages of RNSM with IBR 4-8. However, no evaluation regarding the postoperative pain assessment in RNSM compared with CNSM has been performed.

In this study, a statistical difference was observed in the resting NRS scores at 0-6 postoperative hours between the RNSM and CNSM groups (3.2 ± 1.5 versus 4.2 ± 1.6, respectively; Bonferroni corrected *P* = 0.005); however, which might be subjective depending on the patients, and difference was transient and observed only during the 0-6 postoperative hours. Additionally, no difference was observed at other time periods (PACU, 6-24, and 24-48 postoperative hours), which may suggest that some degree of pain control was achieved through routine IV PCA. This might negate the minor difference in pain associated with skin flap incision and length (4.4 cm versus 8.8 cm; ~4.4-cm difference) between the two types of surgeries. To control for other factors that may affect pain intensity, we only included patients who underwent unilateral NSM and prepectoral IPBR, which involves wrapping the prosthesis in a material such as ADM and placing it behind the skin flap. This approach is less invasive, more cosmetically effective, and less painful since pectoralis major muscle (PMM) dissection is not required. In contrast, subpectoral IPBR involves placement of the prosthetic device in the submuscular pocket behind the PMM 20,21. However, no difference was observed between the two groups regarding acting pain intensity during 48 postoperative hours and the number of patients who required additional analgesics.

Between-group differences were observed in the demographics in terms of incision length, BMI, operation time, and hospital stay. BMI in the RNSM group was lower than that in the CNSM group by 1.7 kg/m^2^ (22.2 ± 3.5 vs. 23.9 ± 3.6 kg/m^2^, respectively), which was statistically, though not clinically, different between the groups, since there was no between-group difference in the breast volume. A smaller incision length, longer duration of operation, and higher medical cost were specific RNSM features, which is consistent with the findings of a previous study 3,8. In this study, the length of hospital stay in the RNSM group was 2 days longer than that in the CNSM group, which might be attributed to the preference of both patients and plastic surgeons for a slightly longer hospital stay in the early phase of RNSM to prioritize safety in an innovative surgical procedure. However, after the initial experience in RNSM, this difference in the length of hospital stay disappeared, as observed in the results of the analysis of length of hospital stay among the last 20 patients in each group (7.8 ± 1.9 versus 7.0 ± 2.2 days, *P* = 0.252). Additionally, there was no between-group difference in the number of patients who presented with PONV.

With regard to postoperative complications, there was no difference between the two groups in the present study, which was consistent with the findings of Lai HW *et al*. 8. However, in a recent report by Lee *et al*., RNSM showed significantly lower postoperative nipple necrosis and high-grade postoperative complications rates, 22 a discrepancy that appears to be due to a difference in the study population. The previous report by Lee *et al*. from our institution included >200 cases of robotic and conventional mastectomy, including autologous breast reconstruction, whereas the current study included only NSM with implant-based reconstruction. All patients who experienced complications in this study recovered completely without any other severe complications.

To our knowledge, this is the first study comparing RNSM versus CNSM with a focus on postoperative pain. However, this study has several limitations, including its retrospective nature, small sample size, and missing information, such as the total consumed dose of analgesics per time unit, frequency and dose of bolus on demand, and chronic pain assessment after 48 hours. We could only evaluate the analgesia quality by comparing the pain intensity based on the NRS scores and the number of patients requiring rescue analgesic as a surrogate. To overcome those limitations and clarify the findings of this study, further large-scale prospective trials are needed. Nonetheless, our findings are clinically significant to the existing literature.

## Conclusions

Despite a statistical difference in the resting NRS scores during the early postoperative phase, the absence of any significant difference in the requirement of additional analgesics between the groups suggested that RNSM does not significantly attenuate postoperative pain intensity in patients with breast cancer without NAC invasion. Further prospective trials are needed to clarify this.

## Figures and Tables

**Table 1:** Clinicopathologic factors of patients selected for the analysis

	RNSM (n = 40)	CNSM (n = 41)	*P* value
**Patients characteristics**			
Age, years	46 ± 8	49 ± 10	0.177
Body mass index, kg/m^2^	22.2 ± 3.5	23.9 ± 3.6	0.030*
**ASA physical status**			0.381
I	19 (48%)	16 (39%)	
II	19 (48%)	19 (46%)	
III	2 (5%)	6 (15%)	
**Smoking history**			0.836
Non-smoker	37 (93%)	36 (88%)	
Ex-smoker	2 (5%)	4 (10%)	
Current-smoker	1 (2%)	1 (2%)	
**Menopause status**			0.268
Premenopausal	28 (70%)	25 (64%)	
Postmenopausal	10 (25%)	14 (36%)	
Perimenopausal	2 (5%)	0 (0%)	
Neoadjuvant chemotherapy	5 (13%)	11 (27%)	0.105
**Pathologic characteristics**			
Pathologic tumor size, cm	1.6 ± 1.3	1.8 ± 1.1	0.420
Multicentric/multifocal lesion	19 (48%)	16 (39%)	0.441
**Lymph node procedure**			0.712
SLNB only	37 (93%)	36 (88%)	
SLNB then ALND	3 (8%)	5 (12%)	
**Pathologic staging, pT**			0.283
Tis	6 (15%)	9 (22%)	
T1	28 (70%)	21 (51%)	
T2	6 (15%)	9 (22%)	
T0	0 (0%)	2 (5%)	
**Pathologic staging, pN**			0.432
N0	37 (93%)	36 (88%)	
N1	2 (5%)	5 (12%)	
N2	0 (0%)	0 (0%)	
N3	1 (3%)	0 (0%)	
**Histopathologic grade**			0.300
G1	13 (33%)	10 (25%)	
G2	23 (57%)	21 (53%)	
G3	4 (10%)	9 (23%)	
**HER2 status**			0.173
Negative	28 (70%)	24 (59%)	
Positive	11 (28%)	11 (27%)	
Not available	1 (3%)	6 (15%)	
**Estrogen receptor status**			0.446
Negative	7 (18%)	10 (24%)	
Positive	33 (83%)	31 (76%)	
**Progesterone receptor status**			0.352
Negative	9 (23%)	13 (32%)	
Positive	31 (78%)	28 (68%)	
**Ki 67**			0.223
Low (<14%)	20 (50%)	26 (63%)	
High (≥14%)	20 (50%)	15 (37%)	

**Notes:** Data are presented as mean ± standard deviation or number of patients (proportion). *P <0.05. **Abbreviation:** RNSM, robotic nipple sparing mastectomy; CNSM, conventional nipple-sparing mastectomy; ASA, American Society of Anesthesiologists; SLNB, sentinel lymph node biopsy; ALNB, axillary lymph node biopsy; HER2, human epidermal growth factor receptor 2; Tis, carcinoma *in situ*.

**Table 2:** Perioperative and surgical characteristics

	RNSM (n = 40)	CNSM (n = 41)	*P* value
Anesthesia time, min	331 ± 74	241 ± 43	<0.001*
Operation time, min	279 ± 63	207 ± 46	<0.001*
**Blood loss**			0.057
≤100 mL	34 (85%)	40 (98%)	
>100 mL	6 (15%)	1 (2%)	
Administered remifentanil (µg/kg/hr)	0.047 ± 0.010	0.047 ± 0.010	0.998
Fentanyl amounts mixed in PCA (µg/kg)	15.0 ± 1.8	14.6 ± 1.6	0.221
Incision length, cm	4.4 ± 0.7	8.8 ± 2.2	<0.001*
Specimen weight, g	388.7 ± 169.5	421.4 ± 176.0	0.400
**Location**			0.144
Right	25 (63%)	19 (46%)	
Left	15 (38%)	22 (54%)	
**Type of reconstruction**			0.195
Direct-to-implant	32 (80%)	37 (90%)	
Tissue expander insertion	8 (20%)	4 (10%)	
Volume of reconstruction implant, mL	366 ± 111	357 ± 110	0.718
**Adjuvant treatment**			
Radiation therapy	5 (13%)	8 (20%)	0.390
Chemotherapy	10 (25%)	12 (29%)	0.666
Endocrine therapy	32 (80%)	26 (67%)	0.180

**Notes:** Data are presented as mean ± standard deviation or number of patients (proportion). *P <0.05. **Abbreviation:** RNSM, robotic nipple-sparing mastectomy; CNSM, conventional nipple-sparing mastectomy; PCA, patient-controlled analgesia.

**Table 3:** Postoperative profile

	RNSM (n = 40)	CNSM (n = 41)	*P* value
Postoperative hospital stays, days	9.2 ± 2.7	7.1 ± 2.0	<0.001*
Postoperative hospital stays, days (recent 20 cases)	7.8 ± 1.9	7.0 ± 2.2	0.252
**Nausea (severe/moderate/mild/none)**			
At PACU	6/2/0/32	5/2/0/34	0.906
0-6 hours	8/5/1/26	10/7/2/22	0.747
6-24 hours	4/6/5/25	7/4/1/29	0.262
Vomiting			
**At PACU**	2 (5%)	3 (7%)	>0.999
0-6 hours	6 (15%)	12 (29%)	0.123
6-24 hours	2 (5%)	6 (15%)	0.264
**Postoperative complication**			
Seroma	2 (5%)	2 (5%)	>0.999
Hematoma	0 (0%)	2 (5%)	0.494
Wound dehiscence	1 (3%)	4 (10%)	0.359
Infection	3 (8%)	3 (7%)	>0.999
**Nipple necrosis**			
Surgical Tx	0 (0%)	1 (2%)	>0.999
Conservative Tx	5 (13%)	5 (12%)	>0.999
**Mastectomy skin flap necrosis**			
Surgical Tx	2 (5%)	6 (15%)	0.264
Conservative Tx	1 (3%)	1 (2%)	>0.999

**Notes:** Data are presented as mean ± standard deviation or number of patients (proportion). *P <0.05. **Abbreviation:** RNSM, robotic nipple-sparing mastectomy; CNSM, conventional nipple-sparing mastectomy; PACU, post-anesthesia care unit; Tx, treatment.

## Abbreviations

NSM: nipple-sparing mastectomy
NAC: nipple-areolar complex
RNSM: Robotic nipple-sparing mastectomy
CNSM: conventional nipple-sparing mastectomy
NRS: numerical rating scale
PONV: postoperative nausea/vomiting
IBR: immediate breast reconstruction
IPBR: immediate prosthesis breast reconstruction
DTI: direct-to-implant
IV-PCA: intravenous patient-controlled analgesia
ADM: acellular dermal matrix
BIS: bispectral index
IV: intravenous
